# Association of fungal secondary metabolism and sclerotial biology

**DOI:** 10.3389/fmicb.2015.00062

**Published:** 2015-02-16

**Authors:** Ana M. Calvo, Jeffrey W. Cary

**Affiliations:** ^1^Department of Biological Sciences, Northern Illinois UniversityDeKalb, IL, USA; ^2^Southern Regional Research Center, United States Department of Agriculture – Agricultural Research ServiceNew Orleans, LA, USA

**Keywords:** secondary metabolism, genetic regulation, sclerotia, VeA, *velvet*, *Aspergillus*, gene cluster, morphogenesis

## Abstract

Fungal secondary metabolism and morphological development have been shown to be intimately associated at the genetic level. Much of the literature has focused on the co-regulation of secondary metabolite production (e.g., sterigmatocystin and aflatoxin in *Aspergillus nidulans* and *Aspergillus flavus*, respectively) with conidiation or formation of sexual fruiting bodies. However, many of these genetic links also control sclerotial production. Sclerotia are resistant structures produced by a number of fungal genera. They also represent the principal source of primary inoculum for some phytopathogenic fungi. In nature, higher plants often concentrate secondary metabolites in reproductive structures as a means of defense against herbivores and insects. By analogy, fungi also sequester a number of secondary metabolites in sclerotia that act as a chemical defense system against fungivorous predators. These include antiinsectant compounds such as tetramic acids, indole diterpenoids, pyridones, and diketopiperazines. This chapter will focus on the molecular mechanisms governing production of secondary metabolites and the role they play in sclerotial development and fungal ecology, with particular emphasis on *Aspergillus* species. The global regulatory proteins VeA and LaeA, components of the *velvet* nuclear protein complex, serve as virulence factors and control both development and secondary metabolite production in many *Aspergillus* species. We will discuss a number of VeA- and LaeA-regulated secondary metabolic gene clusters in *A. flavus* that are postulated to be involved in sclerotial morphogenesis and chemical defense. The presence of multiple regulatory factors that control secondary metabolism and sclerotial formation suggests that fungi have evolved these complex regulatory mechanisms as a means to rapidly adapt chemical responses to protect sclerotia from predators, competitors and other environmental stressors.

## INTRODUCTION

Fungal species are able to develop specialized structures allowing them to disseminate and survive adverse environmental conditions. Aspergilli differentiate by forming conidiophores, structures that produce conidiospores. Some *Aspergillus* species, such as the model species *Aspergillus nidulans*, also produce sexual fruiting bodies known as cleistothecia where meiospores (i.e., ascospores) are generated. Both reproductive processes, asexual and sexual development, are controlled by temporal and spatial genetic regulation (Adams and Yu, 1998; Calvo et al., 2002; Fischer and Kues, 2006). Other species, such as *Aspergillus flavus* or *Aspergillus parasiticus*, form resting structures capable of surviving environmental extremes termed sclerotia that represent vestiges of fruiting bodies incapable of producing ascospores (Coley-Smith and Cooke, 1971; Malloch and Cain, 1972; Wicklow, 1987). Initial evidence presented by Geiser et al. (1996) supported that asexual Aspergilli are often derived from meiotic lineages and postulated for the first time that sclerotia might be vestigial cleistothecia that lost the capacity to produce ascospores. In more recent years, the complementary alpha- and HMG-domain MAT genes have been characterized from *A. flavus* and *A. parasiticus* (Ramirez-Prado et al., 2008). Presence and functionality of mating type genes in *Aspergillus oryzae* was also found, supporting a possible heterothallic breeding system in this fungus (Wada et al., 2012). Furthermore, Horn et al. (2009b, 2014) reported ascospore-bearing ascocarps embedded within sclerotia of *A. flavus* and *A. parasiticus.* The proposed common origin between cleistothecia and sclerotia suggested that conserved genetic regulatory pathways controlling cleistothecia formation could also control sclerotial production. Rapid progress on studies of the cleistothecium-producing model fungus *A. nidulans* and other related fungi [i.e., Dyer and O’Gorman (2012) and references therein] has facilitated uncovering regulatory pathways controlling sclerotial production in other fungi, particularly in *A. flavus*.

Studies have found that a number of genetic regulators controlling the formation of developmental structures, including sclerotia, also govern the production of secondary metabolites (Calvo et al., 2002; Calvo, 2008). While some of these compounds, also termed natural products, are beneficial (e.g., penicillin and lovastatin), other secondary metabolites are deleterious, such as mycotoxins [reviewed in Gloer (2007)]. Among fungal secondary metabolites, aflatoxins (AFs) are probably the most well known and studied. These compounds were discovered after the United Kingdom’s outbreak of Turkey X disease in 1962, caused by consumption of *A. flavus*-contaminated feed and resulted in the deaths of numerous turkey poults (Bennett and Klich, 2003). *A. flavus* is capable of colonizing economically important crops such as peanut, cotton, maize and other oilseed crops both pre- and post-harvest. In the U.S. alone, *A. flavus* costs 100s of millions USD annually due to market losses from AF contaminated crops (Wu, 2004). In addition to AFs, *A. flavus* produces other secondary metabolites and many of them have been found in sclerotia (Gloer, 1995). Genetic regulation of development and secondary metabolism has been intensely studied in the Aspergilli, and in particular *A. flavus* and *A. nidulans*. In this review we focus on the association between secondary metabolism and sclerotial formation in this fungal genus, including genetic co-regulatory patterns leading to the activation of the secondary metabolic gene clusters and formation of sclerotia. Important components of this shared regulatory mechanism are the global regulatory proteins VeA and LaeA, part of the *velvet* complex (Bayram et al., 2008a; Bayram and Braus, 2012). Additionally, we also discuss the possible roles of secondary metabolites associated with sclerotia, particularly in Aspergilli.

## SECONDARY METABOLITES PRESENT IN FUNGAL SCLEROTIA

It is difficult to determine just how many species of fungi exist, but estimates have suggested that the fungal kingdom is very diverse having anywhere from 1.0 to 2.7 million species with only a fraction of these having been isolated and described Hawksworth and Rossman (1997) and Mueller and Schmit (2007). One common theme of many of the described species is that they are prolific producers of biologically active secondary metabolites. The diversity of these natural products rivals that of the fungal kingdom. Fungal secondary metabolites have garnered much attention for their beneficial impact as therapeutic agents (e.g., lovastatin and penicillin) and continue to be mined as a source of important end products and building blocks for pharmaceutical development. On the other hand, secondary metabolites have also received considerable attention for their adverse impact of humans and animals due to their widespread occurrence as mycotoxins (e.g., AFs and fumonisins) on food and feed crops as well as indoor environments. Fungi produce a number of structural classes of secondary metabolites including polyketides (PKs), non-ribosomal peptides (NRPs), hybrid PK-NRPs, indole alkaloids, and terpenes (Keller et al., 2005). In almost all cases the genes responsible for the production of these classes of secondary metabolites are organized as a gene cluster (discussed below). Secondary metabolites of this type that have been identified in sclerotia will be the main focus of this section.

Though many of the recognized biological activities of important secondary metabolites relate to their direct influence on humans and other vertebrates, it is generally accepted that these natural products play key roles in the ecology of the fungus as well. Over the course of evolution, secondary metabolites have been fashioned for numerous biological functions in microorganisms, as chemical messengers between microbes and as a means of defense from predation and competing microbes (Wicklow, 1988; Yim et al., 2007; Rohlfs and Churchill, 2011; Yin et al., 2012). Fungi are much like plants in that; in general, they are static organisms incapable of readily escaping from encroaching predators and competing microbes. In spite of this, fungi are quite successful at inhabiting and surviving for long periods of time in highly competitive environments. It has been hypothesized that these competitive environments have provided considerable selective pressure for fungi to produce an array of antagonistic secondary metabolites as part of their “chemical” defense against numerous fungivores and competitors (Gloer, 2007; Rohlfs et al., 2007; Rohlfs and Churchill, 2011). A recent study showed that arthropod grazing induces a “resistance” phenotype in *A. nidulans* to fungivory that coincided with elevated levels of secondary metabolite and sexual fruiting body formation (Rohlfs and Churchill, 2011). Plants also tend to concentrate secondary metabolites in reproductive structures (e.g., seeds) as a means of defense against herbivores; as well, herbivores tend to avoid feeding or oviposition on plants or plant tissues that contain high levels of secondary metabolites (Rhoades, 1985). In an analogous fashion, various fungi are known to sequester secondary metabolites in asexual conidia and sexual fruiting structures that are critical to survival and which often results in reduced incidences of insect fungivory (Doll et al., 2013).

In addition to conidia and fruiting bodies, numerous fungi also produce structures termed sclerotia. Sclerotia are compacted mats of hyphae produced by certain fungi that allow survival for long periods of time under adverse environmental conditions (Coley-Smith and Cooke, 1971). Upon onset of favorable conditions, sclerotia can germinate to produce large quantities of either hyphae or conidia, and as such they represent a primary source of fungal inoculum in the field. Sclerotia are commonly produced on plant tissues during fungal invasion and eventually end up in soil, or on decaying plant tissues, in the field where they are exposed to predation by insects. In addition to serving as survival structures, in many *Aspergillus* species (e.g., *A. flavus* and *A. nomius*), with proper environmental conditions and mating pair interactions, sclerotia can serve as a substrate (termed stromata) for the formation of sexual structures (Horn et al., 2009a, 2011). The stromata harbor ascospore-bearing cleistothecia, similar to cleistothecia of other ascomycetous species that have a sexual cycle (Horn et al., 2009a, 2014). Many of the genetic mechanisms that connect secondary metabolism to morphogenesis of sexual fruiting bodies have also been shown to control sclerotial production (discussed below). Production of sclerotia represents a substantial metabolic investment by the fungus that is warranted based on the critical role of these structures in reproduction and survival. The importance of sclerotia to fungal biology combined with their high nutrient value to insects would justify the existence of considerable selective pressure on the fungus to produce antiinsectan/antifeedant secondary metabolites as part of their chemical defenses. In fact, this appears to be the case as numerous studies have shown sclerotia to be veritable storehouses of a diversity of secondary metabolites with antiinsectan properties [reviewed in Wicklow (1988) and Gloer (1995, 1997, 2007)]. The fungus’ need for a diverse array of defensive secondary metabolites may be a reflection of the ability of the target organism to develop resistance to specific inhibitory agents. One would predict that the presence of a number of secondary metabolites in sclerotia, many of which may have different modes of action, would make it more difficult for the target organism to evolve resistance either through mutation or acquisition of resistance genes than if it were faced with having to overcome just one inhibitory metabolite.

Perhaps the quintessential example of sclerotia-based chemical defense is that of *Claviceps purpurea*. This ascomycetous fungus produces a group of indole-derived secondary metabolites known as ergot alkaloids (EAs) during growth on a number of plants including many cereal crops (Haarmann et al., 2009). Consumption of food and feeds contaminated with the alkaloid-containing sclerotia (ergot) resulted in vast epidemics of human and animal disease that were reported as early as 600 BC. In addition to *C. purpurea*, a number of chemically diverse EAs are produced by other fungi including many grass endophytes, as well as strains of *Penicillium* and *Aspergillus*, though most of these strains are not known to produce sclerotia (Gloer, 2007). The proposed ecological role of EAs is to protect the fungus by reducing consumption of the host crop by herbivores or from direct consumption by fungivorous insects (Schardl et al., 2006). The remainder of this section will focus on secondary metabolites identified in sclerotia, primarily of *Aspergillus* species, though a few examples will be provided for other fungi. A list of secondary metabolites found in sclerotia from *Aspergillus* species is presented in **Table 1**. Reports by Gloer (1995, 1997, 2007) provide an excellent source of information on the chemistry and biological function of fungal metabolites associated with sclerotia. This review will only touch on new findings since the (Gloer, 2007) publication and provide a few examples of interest. In many cases, previous literature on fungal secondary metabolites describe whole culture extracts and fail to specify if the metabolite(s) was present in sclerotia. In some instances, the investigators report on secondary metabolites that were extracted from isolated sclerotia but fail to indicate if they were also present in other fungal structures such as mycelia and conidia. This review is focused on secondary metabolites of sclerotial origin, but in some cases information will be presented on metabolites that are present in sclerotia as well as other fungal structures, or secreted outside of the cell.

**Table 1:** Secondary metabolites associated with sclerotia of *Aspergillus* species.

Fungus	Metabolite	Structural class	Reference
*A. alliaceus*	Isokotanins	Polyketide	Laakso et al. (1994)
	Nominine	Indole diterpene	Laakso et al. (1994)
	Paspaline	Indole diterpene	Laakso et al. (1994)
*A. arenarius*	Arenarins	Prenylated terphenyl	Oh et al. (1998)
*A. auricomus*	Variecolactol	Sesterterpene lactone	De Guzman et al. (1999)
	Penicillic acid	Polyketide	De Guzman et al. (1999)
	Dihydropenicillic acid	Polyketide	De Guzman et al. (1999)
*A. carbonarius*	Ochratoxin A	Polyketide	Frisvad et al. (2014)
	Carbonarin A	Naphthopyrone	Gloer (1997)
	Aurasperone	Naphthopyrone	Gloer (1997)
*A. flavus*	Aflatoxins	Polyketide	Wicklow and Cole (1982)
	Aflatrems	Indole diterpene	Wicklow and Cole (1982)
	Asparasone	Polyketide	Cary et al. (2014)
	Cyclopiazonic acid	Indole tetramic acid	Wicklow and Cole (1982)
	Aflavarin	Polyketide	Gloer (1995)
	Aflavinines	Indole diterpene	Gloer (1995)
	Aflavazole	Indole diterpene	Gloer (1995)
	Kotanin	Polyketide	Gloer (1995)
*A. leporis*	Leporin A	2-pyridone	Gloer (1995)
*A. melleus*	Bis-indoyl benzenoids	Bis-indoyl benzenoid	Gloer (1995)
	Variecolin	Sesterterpenoid	Gloer (1995)
*A. variecolor*	Variecolin	Sesterterpenoid	Gloer (1995)
*A. nomius*	Nominine	Indole diterpene	Gloer (1995, 1997)
	Aspernomine	Indole diterpene	Gloer (1995, 1997)
	Paspalinine derivatives	Indole diterpene	Gloer (1995, 1997)
*A. ochraceus*	Ochratoxin A	Polyketide	Paster et al. (1984)
	Diketopiperazines	Diketopiperazine	Gloer (1995)
	Ochrindoles	Bis-indoyl benzenoid	Gloer (1995)
*A. sclerotiorum*	Sclerotiamide	Diketopiperazine	Gloer (1997)
	Scleramide	Cyclic hexapeptide	Whyte et al. (2000)
	Oxoasterriquinol D	Bis-indoyl benzenoid	Whyte et al. (2000)
*A. sulphureus*	Penitrem analogs	Indole diterpene	Gloer (1995)
	Radarins	Indole diterpene	Gloer (1995)
	Sulpinines	Indole diterpene	Gloer (1995)
*Aspergillus section Nigri ^a^*	Aflavinines	Indole diterpene	Gloer (1997); Frisvad et al. (2014)
	Ochratoxin A	Polyketide	Frisvad et al. (2014)

*^a^*Aspergillus* section *Nigri* is composed of 15 related black-spored species of Aspergillus.*

One of the most intensely studied fungal genera with respect to production of secondary metabolites is *Aspergillus*. Members of this genus of fungi are ubiquitous in nature and are capable of living as saprophytes in soils or as opportunistic pathogens of humans, plants and animals. With well over 250 identified species of *Aspergillus* (Geiser et al., 2007), probably the best known members of this genus are *A. flavus* and *A. parasiticus,* that produce carcinogenic and toxigenic AFs. Many species of *Aspergillus* produce both sclerotia and the polyketide-derived AFs, however, the majority of the literature has focused on AF production in *A. flavus* as it is most commonly associated with contamination of food and feed crops (Payne and Brown, 1998; Cary et al., 2000; Bhatnagar et al., 2002). AFs are produced during growth of the fungus on oilseed crops such as corn, peanuts, cottonseed, and treenuts and they can also contaminate many additional crops during storage. Production of AFs in *A. flavus* and *A. parasiticus* is known to occur in specialized endosomes of mycelia and subsequently secreted into the environment (Chanda et al., 2009). AFs have also been found in all fungal cell structures including mycelia, conidia, and sclerotia (Wicklow and Cole, 1982; Wicklow and Shotwell, 1983). Though the exact role of AFs in the biology of producing species remains elusive, evidence suggests that they are produced in response to oxidative stress and may also be endowed with antiinsectan properties (Chinnici and Bettinger, 1984; Narasaiah et al., 2006; Grintzalis et al., 2014). In addition, AF production and sclerotial development may be closely related, as increased production of AF precursors was associated with a decrease in sclerotial size (Chang et al., 2002). It was suggested that this may be due to common substrates like acetate being directed toward AF production resulting in lowered availability for biogenesis of sclerotia.

Cyclopiazonic acid (CPA) is an indole-tetramic acid mycotoxin that is produced by a number of species of *Aspergillus* and *Penicillium* (Burdock and Flamm, 2000; Vinokurova et al., 2007). It is a common contaminate of a number of food commodities and is often present as a co-contaminate with AFs (Martins and Martins, 1999). CPA has been found in sclerotia of *A. flavus*, however, it was also detected in mycelia (Wicklow and Cole, 1982). Though its role in the ecology of the fungus is not known, CPA has been shown to be an inhibitor of calcium-dependent ATPase in the sarcoplasmic reticulum with exposure in some animals leading to organ necrosis and death (Riley et al., 1992).

Another mycotoxin of importance to human health is ochratoxin A (OTA; El Khoury and Atoui, 2010). OTA is produced by several species of *Aspergillus* and *Penicillium* via a pentaketide that is derived from a dihydrocoumarin coupled to β-phenylalanine. It is detected worldwide in various food and beverage sources. OTA can have several toxicological effects such as nephrotoxic, hepatotoxic, neurotoxic, teratogenic, and immunotoxic. OTA has been isolated from sclerotia of *Aspergillus ochraceus*, *Aspergillus sclerotioniger*, and *Aspergillus carbonarius*, with OTA isolated from the latter shown to have antiinsectan properties (Paster et al., 1984; Wicklow et al., 1996; Frisvad et al., 2014). Only a few strains of *Aspergillus* section *Nigri* have been reported to produce sclerotia, but when cultured in artificial media supplemented with natural substrates such as fruits and grains, sclerotial production was induced along with numerous sclerotial secondary metabolites (Frisvad et al., 2014). In addition to detection of OTA, some isolates were found to produce apolar indoloterpenes of the aflavinine type and okaramines (Frisvad et al., 2014; Petersen et al., 2014).

A number of fungi produce polyketide-derived melanins which are the black or near-black pigments formed by oxidative polymerization of phenolic compounds produced by the dihydroxynaphthalene (DHN)-melanin pathway (Wheeler, 1983; Butler and Day, 1998). Melanin has been shown to be a virulence factor in plant, animal, and human pathogenic fungi and it also functions in survival and longevity in nature of fungal propagules such as sclerotia (Butler and Day, 1998). Sclerotial DHN-melanins have been reported as a component of black sclerotia of *Sclerotinia sclerotiorum* and *S. trifoliorum* (Butler et al., 2009). Recently, an *A. flavus* gene cluster was found to be responsible for the production of a sclerotia-specific pigment identified as the polyketide, asparasone (discussed below; Cary et al., 2014). Sclerotia produced by mutants of the asparasone polyketide synthase (PKS) lacked dark pigmentation, were significantly less resistant to insect predation than wild-type sclerotia and were more susceptible to the deleterious effects of ultraviolet light and heat. Fungal sclerotia and conidia were previously thought to be mostly resistant to this type of damage due to the presence of DHN-melanins. The study of Cary et al. (2014) showed that the dark brown pigments in *A. flavus* sclerotia derive from anthraquinones produced by the asparasone cluster rather than from the typical DHN-melanin pathway.

## GLOBAL GENETIC REGULATORY MECHANISMS GOVERNING PRODUCTION OF SECONDARY METABOLITES THAT INFLUENCE SCLEROTIA

The global regulatory proteins VeA and LaeA, components of the *velvet* nuclear protein complex, control both development and secondary metabolism in numerous fungi, including *Aspergillus* species. The characterization of the *veA* gene began more than 60 years ago, when Kafer (1965) generated the first *veA* mutant in *A. nidulans*, *veA1*, a mutant with partial loss-of-function. However, its characterization was delayed for many years due to the fact that the VeA predicted protein did not demonstrate homology with any other proteins of known function. Further studies with *veA* deletion mutants in *A. nidulans* and in other fungi provided valuable insight into the role of this regulator. *veA* is known to have a role in activating sexual development and inhibiting asexual development (Champe et al., 1981; Yager, 1992; Kim et al., 2002). Interestingly the role of *veA* in the regulation of morphogenesis is light-dependent; light reduces and delays cleistothecial formation and promotes conidiation in *A. nidulans* strains with a *veA* wild-type allele, while in the dark the fungus develops fruiting bodies (Yager, 1992; Stinnett et al., 2007). Deletion of *veA* in *A. nidulans* resulted in hyperconidiating strains unable to produce cleistothecia (Kim et al., 2002; Kato et al., 2003). Similarly, deletion of *veA* increases conidiation and completely blocks sclerotial formation in *A. flavus* and *A. parasiticus* (Calvo et al., 2004; Duran et al., 2007).

Another major breakthrough contributing to the understanding of *veA* function was the discovery of its regulatory role in secondary metabolism in *A. nidulans* (Kato et al., 2003). *veA* was shown to control the biosynthesis of several compounds including antibiotics and mycotoxins, specifically sterigmatocystin (ST), the penultimate intermediate in the AF biosynthetic pathway (Kato et al., 2003). Further studies revealed this regulatory role to be conserved in many other fungi. Importantly, *veA* was demonstrated to be required for the production of AFs in *A. parasiticus* and *A. flavus*, as well as CPA and aflatrem in *A. flavus* (Calvo et al., 2004; Duran et al., 2007, 2009). Studies of the *veA*-dependent transcriptome in *Aspergillus fumigatus* indicated that *veA* affects the expression of 100s of genes (Dhingra et al., 2013), while studies in *A. flavus* and * Fusarium verticillioides* demonstrated a role for *veA* in response to oxidative stress (Baidya et al., 2014; Lan et al., 2014) and hydrolytic activity (Duran et al., 2014). However, in this review we will mainly focus on the role of *veA* and known *veA*-related regulatory factors in the control of morphogenesis, particularly in the formation of sclerotia and in the biosynthesis of secondary metabolites.

**FIGURE 1:**
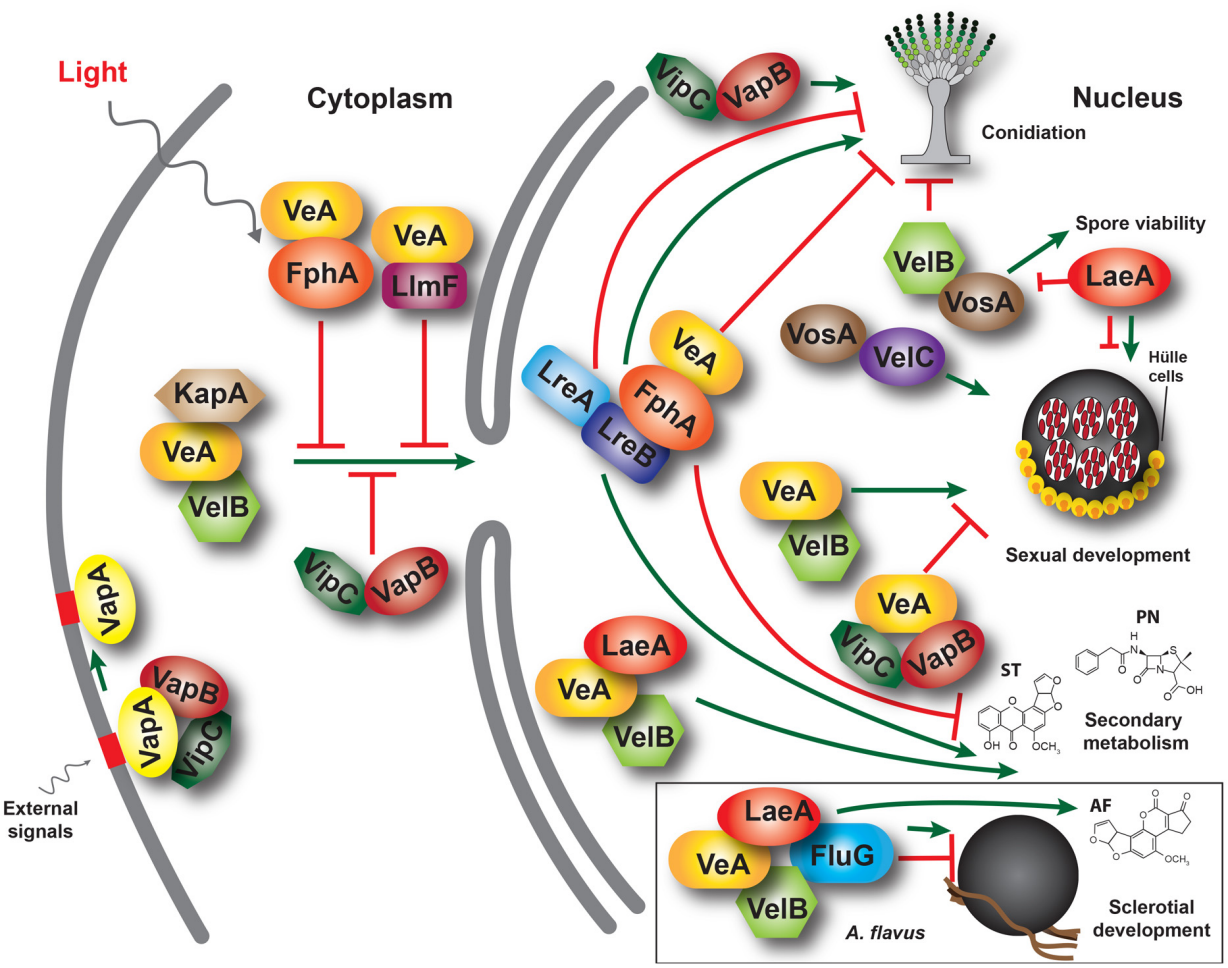
**A model illustrating interactions between *velvet* family proteins, LaeA and other associated proteins in the model fungus *Aspergillus nidulans*.** The α-importin KapA transports the VeA-VelB dimer from the cytoplasm to the nucleus, particularly in the dark. This transport is negatively influenced by other proteins, such as FphA, LlmF and Vip-VapB dimer in the light. In the nucleus, VelB-VeA activates sexual development and can interact with LaeA, forming the *velvet* complex. VeA also interacts with FphA, which is associated with LreB-LreA forming a light-sensing protein complex. VelB, repressor of asexual development, also forms homodimers and heterodimers with VosA, a protein required for spore viability activating trehalose biosynthesis. VosA also interacts with VelC, which positively regulates sexual development. Additionally, VipC and VapB associate with VeA in the nucleus repressing cleistothecial formation. These complexes regulate development and secondary metabolism in a coordinated manner. VeA, LaeA, and VelB have also been shown to control sclerotial and AF production in *A. flavus*, where they also form a protein complex, together with FluG (box). ST, sterigmatocystin. PN, penicillin; AF, aflatoxin B1.

Numerous putative *veA* homologs have been identified in other fungal species and many of them have been experimentally characterized (Li et al., 2006; Dreyer et al., 2007; Bayram et al., 2008b; Chettri et al., 2012; Dhingra et al., 2012; Laskowski-Peak et al., 2012; Myung et al., 2012; Lopez-Berges et al., 2013). In *A. nidulans* the study of possible *veA*-interacting proteins revealed that VeA forms complexes with other proteins (Bayram et al., 2008a; Calvo, 2008; Purschwitz et al., 2008; **Figure 1**). After its transport to the nucleus by the alpha-importin KapA (Stinnett et al., 2007; Araujo-Bazan et al., 2009) VeA forms a complex with the red phytochrome FphA (Purschwitz et al., 2008). This interaction is dependent on the presence of the tetrapyrrole chromophore. LreA and LreB, blue sensing proteins, do not interact directly with VeA, but through FphA association; the FphA protein was found to interact with LreB, which interacts with LreA. Deletion of either *fphA* or *lreA*/*lreB* genes affected sexual development as well as secondary metabolism in *A. nidulans* where they play antagonistic functions (Purschwitz et al., 2008). FphA also negatively affects VeA transport to the nucleus in the presence of light. It is likely that a similar regulatory output of the light-sensing proteins is also conserved in *A. flavus*.

Additional studies in *A. nidulans* showed that VeA also interacts with LaeA, forming part of the *velvet* complex (Bayram et al., 2008a). LaeA encodes a putative methyl transferase involved in chromatin remodeling (Keller et al., 2005; Bok et al., 2006b; Reyes-Dominguez et al., 2010). In addition, LaeA influences VeA post-translational modifications and inhibits sexual development in *A. nidulans* in response to light (Sarikaya Bayram et al., 2010). Moreover, *laeA* has been shown to be a positive regulator of gene clusters involved in secondary metabolism in this model organism (Keller et al., 2005; Bok et al., 2006a). In *A. flavus* the *laeA* homolog is necessary for production of AFs and sclerotial formation (Kale et al., 2008). Additionally, it has been shown that *laeA* is a negative regulator of *veA* expression in *A. flavus*. Transcriptome analysis of *A. flavus* wild-type and *laeA* deletion strains indicated that *laeA* not only regulates AF production but also controls the expression of other secondary metabolic gene clusters (Georgianna et al., 2010). Similar to FphA, an *A. nidulans* LaeA-like putative methyltransferase, designated LlmF, also interacts with VeA, negatively affecting VeA transport to the nucleus and acting as negative regulator of ST production and sexual development (Palmer et al., 2013).

Another component of the *velvet* complex interacting directly with VeA is VelB (Bayram et al., 2008a; Park et al., 2012), a member of the *velvet* protein family together with VosA and VelC (Ni and Yu, 2007; Park et al., 2014). In *A. nidulans*, VelB binds to VeA in the cytoplasm and they are co-transported to the nucleus (Bayram et al., 2008a). Similar to the *veA* deletion mutant, deletion of *velB* results in a strain unable to display a light-dependent developmental pattern and it is unable to form cleistothecia (Bayram et al., 2008a). However, dissimilar from the *veA* deletion, absence of *velB* only showed reduced and delayed production of ST. VelB also interacts with VosA (Bayram et al., 2010). The *velvet* domain in these two proteins has been shown to bind DNA in *A. nidulans* (Ahmed et al., 2013) and in *Histoplasma capsulatum*, where there are involved in the activation of the yeast-phase specific gene expression program (Beyhan et al., 2013). In addition, the VelC *velvet* protein functions as a positive regulator of sexual development in *A. nidulans* (Park et al., 2014). Homologs of *A. nidulans* VelB, and VelC have also been characterized in *A. flavus* (Chang et al., 2013). Deletion of *A. flavus velB* (but not *velC*), similar to the case of *veA* (Duran et al., 2007), prevents sclerotial formation and AF biosynthesis.

In addition to the interaction between *A. flavus* LaeA and VelB with VeA, Chang et al. (2013) also described interactions between LaeA and VelB with FluG (**Figure 1**), a known developmental regulator previously characterized in *A. nidulans*. FluG contributes to the inactivation of the FadA G-protein signaling pathway in the model fungus, leading to ST production and allowing sexual and asexual development. Mutations in *A. nidulans fluG* result in proliferation of undifferentiated vegetative hyphae that yield fluffy cotton-like colonies lacking the capacity to produce ST (Adams et al., 1992; Wieser et al., 1994). FadA function was also conserved in the AF-producer *A. parasiticus* (Hicks et al., 1997). Evidence for a connection between *fluG* and *veA* was previously provided by Mooney and Yager (1990) and Yager et al. (1998). Mooney et al. (1990) found three extragenic *veA1* suppressor mutations that restored light-dependent conidiation in *A. nidulans* corresponded to different *fluG* alleles. This suggested that *veA* light-dependent activities are related to *fluG* function. *fluG* is involved in the synthesis of a diffusible compound that triggers the FluG signaling pathway directing conidiation and mycotoxin biosynthesis while reducing vegetative growth (Lee and Adams, 1996). The diffusible molecule was determined to be dehydroaustinol (Rodriguez-Urra et al., 2012). Two gene clusters in *A. nidulans* have been found to encode the complete dehydroaustinol pathway (Lo et al., 2012). However, co-culturing experiments did not show a similar diffusible secondary metabolite produced by *A. flavus*. These results suggest that the function of *fluG* and the signaling pathways related to conidiation might be different in these two related Aspergilli (Chang et al., 2012). Based on *A. flavus* studies, Chang et al. (2013) postulated that a delicate balance in the interaction between VeA, VelB, FluG, and LaeA is necessary to maintain normal sclerotiogenesis, conidiogenesis and secondary metabolism, where FluG plays an antagonistic role with respect to VeA, VelB, and LaeA regarding sclerotia formation (Chang et al., 2012). Deletion of *fluG* resulted in a notable increase in sclerotial formation but did not affect AF production. This also differs from the role of *fluG* in *A. nidulans*, where this gene is necessary for ST biosynthesis.

Other characterized VeA-interacting proteins include VipC-VapB methyltransferases, released from the VapA-VipC-VapB membrane-bound complex (Sarikaya Bayram et al., 2014). Presence of VipC-VapB reduces the abundance of the nuclear VelB-VeA-LaeA complex resulting in decreased sexual development. Additionally, VapB also decreases histone 3 lysine 9 trimethylation favoring asexual development.

Post-translational modification of VeA, such as that detected in LaeA-dependent modification in *A. nidulans*, could also have an effect on the *velve*t complex function (Sarikaya Bayram et al., 2010). Purschwitz et al. (2009) demonstrated that VeA is phosphorylated. Later Bayram et al. (2012) showed that MpkB phosphorylates VeA. The MAP-kinase *mpkB*, homolog of *FUS3* in *Saccharomyces cerevisiae*, was first characterized in *A. nidulans* by Paoletti et al. (2007) and Atoui et al. (2008). MpkB transcription increased during sexual development and deletion of the *mpkB* gene resulted in sterility (Paoletti et al., 2007), as well as in a decreased in the expression of ST biosynthetic genes and concomitant ST biosynthesis (Atoui et al., 2008). Furthermore, the absence of *mpkB* also decreased the expression of genes in the penicillin and terrequinone A gene clusters (Atoui et al., 2008). *mpkB* is also necessary for normal expression of *laeA*, that as discussed above, is a global regulator of secondary metabolism (Atoui et al., 2008). The *mpkB* homolog is present in the *A. flavus* genome, however, its possible function in sclerotial development and secondary metabolism has not yet been characterized experimentally in this AF producer.

Both sclerotial and conidial development and secondary metabolism have been shown to be modulated by *A. flavus* oxylipins as well as by endogenous plant oxylipins that interact with the infecting fungus (Burow et al., 1997; Calvo et al., 1999; Brown et al., 2008; Affeldt et al., 2012; Scarpari et al., 2014). The *A. flavus* genome harbors four dioxygenase genes, *ppoA*, *ppoB*, *ppoC*, and *ppoD*, and one lipoxygenase gene, *loxA* (Brown et al., 2008, 2009). In the model fungus *A. nidulans* it has been shown that *veA* is important for *ppo*-dependent regulation of development. For instance, *veA* regulates * ppoA* expression (Tsitsigiannis et al., 2004). Furthermore, the triple mutant *ppo*A/B/C showed an increase in *veA* expression suggesting a regulatory loop between *ppo* genes and the master regulator *veA* (Tsitsigiannis et al., 2005). Absence of these genes results in alteration in morphological and chemical development in *A. flavus* [review by Amaike and Keller (2011)]. For example, strains with deletion of these five genes showed high levels of AF production and sclerotial formation (Brown et al., 2009). The antagonistic roles of different types of oxylipins appear to contribute to a balance between conidiation and sclerotial formation.

The necessity of *veA* for sclerotial production and AF biosynthesis could also be related to the requirement of *veA* for a proper oxidative stress response in *A. flavus* (Baidya et al., 2014). Using modulators that inhibit oxidative stress as well as thiol redox state, Grintzalis et al. (2014) demonstrated that oxidative stress regulates both AF biosynthesis and sclerotial development. Several research groups have also provided evidence of the association between AF production and oxidative stress in Aspergilli (Chang et al., 2011; Reverberi et al., 2012; Hong et al., 2013; Roze et al., 2013).

**FIGURE 2:**
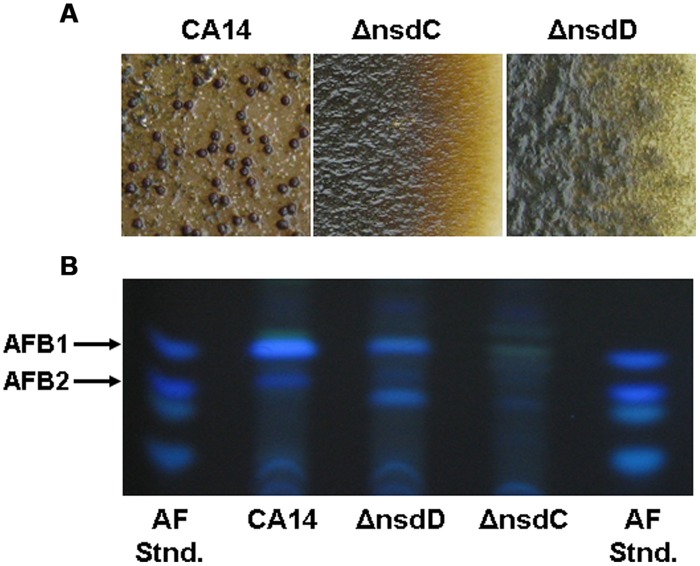
**Production of sclerotia and aflatoxins in *A. flavus* CA14 Δ*nsdC* and Δ*nsdD* mutants. (A)** Surface of colonies demonstrating sclerotial production after 14 days growth in the dark. Sclerotia were absent in the Δ*nsdC* and Δ*nsdD* mutants and were produced in the wild-type CA14 (dark structures). **(B)** TLC analysis of aflatoxin production from the wild-type CA14, Δ*nsdC,* and Δ*nsdD* mutants. Extracts (5 ul) were spotted onto 250 um silica gel TLC plates and metabolites were separated in ethyl acetate: methanol: water (40:1:1). Aflatoxin standards were also spotted on the plate. Adapted and modified from Cary et al. (2012).

Recently other regulatory genes have been found to affect development and secondary metabolism in *A. flavus*, specifically *nsdD* and *nsdC* (Cary et al., 2012). The * nsdD* gene, first described in *A. nidulans*, encodes a GATA-type zinc finger transcription factor necessary for cleistothecia formation (Han et al., 2001), while *nsdC* encodes a C_2_H_2_ zinc finger-type transcription factor shown to negatively regulate asexual sporulation (Kim et al., 2009). *veA* only slightly affects *nsdD* expression (Kato et al., 2003), and has no effect on *nsdC* expression (Kim et al., 2009), suggesting that the role of these genes is independent of *veA* in *A. nidulans*. In *A. flavus*, both *nsdC* and *nsdD* are necessary for sclerotial production and normal levels of AF biosynthesis (**Figure 2**; Cary et al., 2012).

Functional genomic analysis is a powerful approach that has helped to elucidate the genetic connections between sclerotial formation and secondary metabolism. For instance, Wu et al. (2014) compared the transcriptome of mycelium and sclerotium developmental stages and found that backbone genes of 38 secondary metabolite pathways were transcribed in both the mycelial and sclerotial cultures, including the AF biosynthetic pathway. A transcriptome study by Lin et al. (2013) of *A. flavus* cultures treated with 5-azacytidine, an inactivator of DNA methyltransferase, provided further evidence that secondary metabolism and development are co-regulated. Addition of 5-azacytidine altered the expression of backbone genes of two identified secondary metabolite gene clusters, #35 and also #27, both of which have been demonstrated experimentally to be associated to sclerotial biology in either a *veA-* or *laeA*-dependent manner (Forseth et al., 2012; Cary et al., 2014). Additionally, Chang et al. (2014) studied the transcriptome of cultures treated with decanal and observed that this volatile compound halted development at the vegetative state rendering the fungus unable to produce sclerotia. This coincided with early transcriptional activation of AF and kojic acid biosynthesis gene clusters as well as subtle altered timing of other secondary metabolite gene clusters.

## GENE CLUSTERS PRODUCING SECONDARY METABOLITES ASSOCIATED WITH SCLEROTIA

Rapid progress in sequencing of fungal genomes, coupled with bioinformatics, has provided researchers with an *in silico* approach for identifying potential secondary metabolic gene clusters (Bergmann et al., 2007; Winter et al., 2011; Ehrlich and Mack, 2014). Many of the prediction algorithms (e.g., SMURF, antiSMASH, and MIDDAS-M) in use are based on identification of core or “backbone” genes that encode enzymes such as a PKSs, NRPSs, or dimethylallyltryptophans (DMATs) as well as closely allied genes encoding “decorating” enzymes (e.g., dehydrogenases, methyltransferases, and oxidases), transcription factors and transporters (Khaldi et al., 2010; Medema et al., 2011). The MIDDAS-M algorithm has been used to identify potential secondary metabolic gene clusters that may not contain common core genes such as that for ustiloxin B, an *A. flavus* secondary metabolite produced by a ribosomal peptide synthetic pathway (Umemura et al., 2014). These types of algorithms have resulted in the identification of numerous putative secondary metabolic gene clusters in fungi, typically between 30 and 40 in *Aspergillus* species sequenced (Brakhage and Schroeckh, 2011; Andersen et al., 2013; Inglis et al., 2013) including as many as 55 in *A. flavus* (Georgianna et al., 2010). Some of the products of these clusters have been verified based on prior knowledge of genes and metabolites that constitute the cluster (e.g., AF and CPA). In other cases, the products have been predicted in one species based on homology to genes known to produce the metabolite in another fungal species; for example, the identification of the penicillin gene cluster in *A. flavus* based on amino acid identity to the known penicillin biosynthetic genes from *A. nidulans*. However, in most instances, the identity of the metabolites encoded by predicted secondary metabolic gene clusters remains unknown. In these instances the clusters have been termed “orphans.” In a number of cases, these orphan clusters can be “cryptic” or silent when the conditions required to activate expression of the cluster genes have not been determined (Brakhage and Schroeckh, 2011; Brakhage, 2013). Once a putative secondary metabolic gene cluster has been identified, a number of techniques can then be utilized to aid in identification of the cluster metabolite (Brakhage and Schroeckh, 2011; Sanchez et al., 2012).

When genes from orphan clusters are actively transcribed under laboratory growth conditions, standard gene-inactivation techniques can be applied, coupled with comparative metabolic profiling of the pathway mutant and the control strain using LC–MS. A common method used for the identification of cryptic gene cluster metabolites is to overexpress the pathway-specific transcriptional activator (if known) by placing it under the control of a strong inducible or constitutive promoter. For example, normally silent genes of the *A. nidulans* aspyridone (apd) gene cluster were activated by coupling of the *apdR* transcriptional activator to the inducible alcohol dehydrogenase promoter of *A. nidulans*, thus allowing identification of aspyridones A and B (Bergmann et al., 2007). In the absence of a pathway-specific transcription factor, it may be possible to activate gene expression of cryptic clusters by overexpressing global regulatory factors. This is exemplified by the use of overexpressing and deletion mutants of the global regulator, *laeA*, to identify the terrequinone A gene cluster in *A. nidulans* (Bok et al., 2006a). In keeping with epigenetic regulation of secondary metabolite production, a number of chemical agents (e.g., histone deacetylase or DNA methyltransferase inhibitors) or genes (e.g., inactivation of a histone deacetylase or sumoylation gene) that modulate chromatin structure have been used to successfully induce expression of cryptic clusters [reviewed in Sanchez et al. (2012) and Brakhage (2013)].

A recent study indicated the presence of secondary metabolite-mediated crosstalk between two separate gene clusters (Forseth et al., 2012). Comparative metabolomics of gene knockout, knockdown (RNAi-based), and overexpression strains of *A. flavus* were used to identify a group of secondary metabolites derived from two *laeA*-regulated orphan gene clusters, designated lna and lnb. The lna cluster is located on chromosome 6 and lnb on chromosome 8. The two clusters harbor non-canonical NRPS genes (*lnaA* and *lnbA*) with high sequence identity as well as associated genes encoding tailoring enzymes that are involved in the production of a group of piperazines. It was shown that addition of the one of the piperazine metabolites, produced almost exclusively by the lnaA cluster, to wild-type cultures greatly increased expression of the lnaB NRPS. The apparent “sensing” of a metabolite produced by a separate but related gene cluster may represent another layer in the complex regulation of secondary metabolism in fungi. Interestingly, loss of these *lnaA-* and *lnaB*-derived piperazines resulted in a significant reduction in sclerotial formation in the mutant strains, thereby demonstrating a role of these secondary metabolites in fungal development.

Lastly, the most ecologically based of all secondary metabolite induction techniques, is the simulation of interactions in nature between the fungus and other resident microbes. This technique is based on the premise that microorganisms share ecological niches; and as such, produce secondary metabolites as a means of intra- and interspecies communication or as defense mechanisms. By simulating these interactions in culture, using two or more organisms, there is a chance that the organism of interest will respond by eliciting production of a secondary metabolite. For example, utilizing microarray technology with co-cultivation techniques, the interaction of *A. nidulans* with the soil-dwelling actinomycete, *Streptomyces rapamycinicus*, induced the expression of a cryptic gene cluster in *A. nidulans* involved in the production of the polyketide, orsellinic acid (Schroeckh et al., 2009).

Sclerotia represent a means by which fungi maintain a quiescent viable state in the absence of a suitable host or of conditions favoring active growth (Coley-Smith and Cooke, 1971). As such, mature sclerotia are essentially dormant metabolically, and therefore would not be amenable to any of the methods discussed above for activation of cryptic secondary metabolic pathways. However, it is probable that many of the secondary metabolites present in sclerotia are produced in the hyphae that coalesce during the early phases of sclerotial morphogenesis. Most sclerotial metabolites identified so far in fungi have been identified from extracts of sclerotia generated under laboratory conditions on artificial media. It is likely that sclerotia found in nature harbor many additional as of yet unidentified secondary metabolites. Below we describe a number of genetically and biochemically well characterized secondary metabolite gene clusters whose products have been found in sclerotia of filamentous fungi. As most of these clusters have been thoroughly reviewed in the literature, only a brief synopsis with references will be provided here.

### ERGOT ALKALOIDS

Ergot alkaloids represent a complex family of indole derivatives with diverse structures and broad biological and pharmacological activities. The genetics and enzymology of EA biosynthesis is detailed in reviews by Wallwey and Li (2011) and Jakubczyk et al. (2014). Chemically, EAs can be divided into three groups: ergoamides, ergopeptines, and clavines. The biosynthetic gene clusters responsible for the production of each of these types of EAs have been identified in a number of fungal species. The gene cluster in *C. purpurea* leading to the formation of complex ergopeptines consists of 14 genes spanning about 68.5 kb of the genome. The *Claviceps fusiformis* cluster is responsible for the production of the clavines, agroclavine and elymoclavine, that lack the peptide moieties present on the ergoline ring of ergopeptines. The *C. purpurea* and *C. fusiformis* gene clusters are homologous with the exception of three genes that are lacking in *C. fusiformis*. These genes (*lpsA1, lpsA2,* and *lpsC*) encode NRPSs that are responsible for biosynthesis of the peptide moieties present in the ergopeptines. *A. fumigatus* also produces the clavine-type metabolites, fumigaclavines, but these have only been associated with conidiation. No genes encoding a putative pathway-specific transcriptional activator or transporter have been identified in EA gene clusters.

The genes involved in EA biosynthesis are divided into early and late pathway steps. The first step of the early pathway is catalyzed by the dimethylallyl prenyltransferases (DmaW) that prenylates L-tryptophan in the presence of dimethylallyl pyrophosphate (DMAPP) to form DMAT. Subsequent methylation (EasF) and two successive oxidations (EasC and EasE) produce chanoclavine-I, the ergoline ring C structure. Chanoclavine-I is then oxidized by EasD to generate the aldehyde form which in *Claviceps* is subsequently cyclized by EasA and EasG reductases to form the unsaturated ergoline ring D structure, agroclavine, that represents the last intermediate of the early pathway. The late step pathway genes encode an oxidase (CloA) responsible for the formation of paspalic acid which, either spontaneously or via an isomerase, forms lysergic acid. Three NRPSs (Lps1, Lps2, and lpsC) activate lysergic acid and form the tripeptide moiety of the ergopeptine end products.

### ASPARASONE A

**FIGURE 3:**
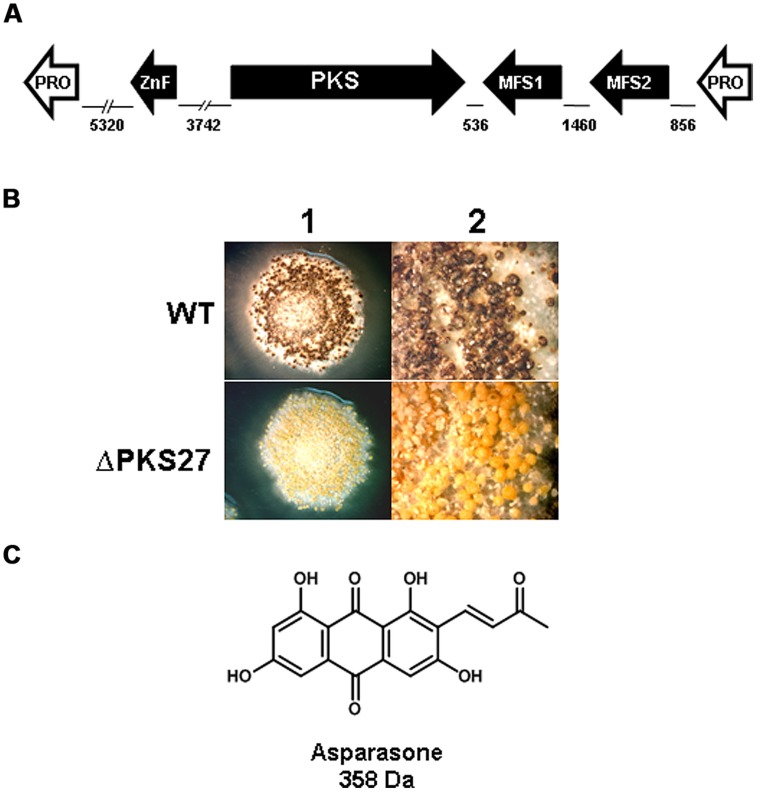
**(A)** Schematic representation of the *A. flavus* asparasone gene cluster. The cluster is composed of four genes [putative Gal4-type transcription factor (ZnF); polyketide synthase (PKS); putative high-affinity glucose transporter (MFS1); and a second putative MFS transporter (MFS2)]. The two hypothetical protein encoding non-cluster genes (Pro) flanking the asparasone cluster are shown in white. The size in bp of intergenic regions are shown. Arrowheads denote direction of transcription. **(B)** Microscopic examination of sclerotia. Sclerotium production in Af70 pyrG-1 (WT) and an Af70 Δ*pks27* mutant was observed using an Olympus SZH10 stereomicroscope and Nikon DS-Qi1 camera. Panel: (1) colony surface, X10 magnification; (2) colony surface, X25 magnification. **(C)** Chemical structure of asparasone A. Adapted and modified from Cary et al. (2014).

Expression of genes present in an *A. flavus* cluster, designated #27 based on SMURF analysis by Georgianna et al. (2010) was found to be significantly downregulated in a *veA* mutant (Cary et al., 2014). The cluster was predicted to consist of a Zn(2)-Cys(6)-type transcription factor, PKS, two putative transporters and a gene encoding a hypothetical protein. A schematic depiction of the cluster is shown in **Figure 3A**. Expression of the *pks27* gene was first observable at 48 h, was maximal at 120 h, and decreased by 144 h (Cary et al., 2014). Transcription paralleled sclerotial development and pigmentation which appeared to be maximal at 120 h in wild-type *A. flavus*. The transcription factor, *znf27,* was required for wild-type levels of expression of the other three cluster genes but not for the gene encoding the hypothetical protein. The putative high-affinity glucose (*mfs1*) and MFS transporter (*mfs2*) genes showed an expression profile similar to that observed for the *pks27* and the *znf27* genes. qRT-PCR of RNA isolated from mycelia, conidia, and sclerotia of the *A. flavus* wild-type showed that expression of *pks27* and *znf27* was specific to the sclerotium.

Inactivation of *pks27* resulted in *A. flavus* colonies that produced only grayish-yellow sclerotia, instead of the characteristic dark brown color of the wild-type, indicating that the mutational defect was only in pigmentation and not in sclerotial maturity (**Figure 3B**). Comparison of extracts of the wild-type and the *Δpks27* mutant by ultra-high performance liquid chromatography and mass spectrometry revealed a metabolite of mass 358 Da that was present in the wild-type but missing in the mutant. Based on this mass, the metabolite was putatively identified as the anthraquinone asparasone A [1,3,6,8-tetrahydroxy-2-(1′-hydroxy-3′-oxobutyl)-anthraquinone; *M* = 358 Da; **Figure 3C**]. It was previously reported to be produced by *A. parasiticus* which is a close relative of *A. flavus* (Sobolev et al., 1997). The identification of the 358 Da metabolite as asparasone A was confirmed by LC–MS comparison with an authentic asparasone A standard. It was hypothesized that dehydration of asparasone would result in conjugated olefins which, like styrene, might rapidly polymerize in the presence of metal dioxygenases such as laccases to form the dark sclerotial pigment (Cary et al., 2014).

### AFLATOXIN

The genetics, molecular biology, and biochemistry of AF biosynthesis in *A*. *flavus* and *A. parasiticus* have been the focus of a number of reviews, and we direct the reader to these references for more detailed information (Yu et al., 2004; Ehrlich et al., 2005; Georgianna and Payne, 2009; Yu, 2012). Many of the contributions to our understanding of AF biosynthesis and its regulation have come from studies in the model fungus, *A. nidulans*, on the biosynthesis of ST. ST a precursor of AF, and the genes for ST biosynthesis in *A. nidulans* are highly homologous to those required for the production of ST in the AF gene cluster. The AF biosynthetic gene cluster of *A. flavus* spans ∼70 kb of chromosome 3 and consists of 28 genes including two regulatory factors, *aflR* and *aflS* (*aflJ*). AflR is a Zn(2)-Cys(6)-type, pathway-specific transcriptional activator while AflS does not share any significant identity with other fungal proteins but has been shown to be required for AF production. AflS has been shown to interact with AflR and facilitate the activation of other AF biosynthetic genes (Du et al., 2007). AflR protein binds to the palindromic sequence 5′-TCGN_5_CGA-3′ (or deviations thereof) found in the promoter region of all AF biosynthetic genes. The AF gene cluster in *A. flavus* is under the control of the global regulators, VeA, NsdD, NsdC, and LaeA (Duran et al., 2007; Kale et al., 2008; Cary et al., 2012).

Aflatoxins are a group of polyketide-derived furanocoumarins that are produced from acetate via a PKS (AflC) and two fatty acid synthetases (AflA and AflB), and a number of tailoring enzymes. AF biosynthesis requires at least 18 known enzymatic reactions to catalyze synthesis of the four major AFs, AFB1, AFB2, AFG1, and AFG2 found in *A. parasiticus*. In general, *A. flavus* only produces AFB1 and AFB2. Just outside of the distal end of the AF gene cluster in *A. parasiticus* and *A. flavus* is a conserved sugar utilization gene cluster. However, the genetic composition at the proximal end (the end closest to the telomere) of the AF cluster is not conserved in these two species. *A. flavus* strains contain a deletion at the proximal end of the cluster that result in functional loss of *aflU* (*cypA*) and *aflF* (*norB*) genes. The inability of *A. flavus* to produce the G toxins is due to the partial deletions of *aflU* and *aflF*, which encode a P450 monooxygenase and a putative aryl alcohol dehydrogenase, respectively, and are required for conversion of hydroxymethyl-ST to AFG1 in *A. parasiticus* (Zeng et al., 2011).

*Aspergillus flavus* as a species contains two morphotypes that differ in sclerotial size and in their ability to produce AFs. Large (L) and small (S) sclerotial strains are often found in soils from agricultural fields, and the S strains are generally found to produce higher levels of AF than L strains (Zhang and Cotty, 2006; Horn, 2007). *A. flavus* is a genetically diverse species and, unlike other aflatoxigenic *Aspergillus* species, a portion of *A. flavus* populations has lost the ability to produce AFs (Cotty and Bhatnagar, 1994). A survey of 38 non-aflatoxigenic *A. flavus* strains, isolated from across the Southern United States, identified eight patterns of gene deletion present in the AF gene cluster (Chang et al., 2005). There is evidence that gene loss in the AF gene cluster of non-aflatoxigenic *A. flavus* isolates is irreversible, and that balancing selection maintains non-aflatoxigenicity and lineage-specific gene loss in *A. flavus* populations (Moore et al., 2009, 2011).

### AFLATREMS

Both aflatrem and its isomer, β-aflatrem (502 Da), are indole-diterpenes that have been isolated from the sclerotia of *A. flavus* (TePaske et al., 1992). Aflatrems are tremorigenic mycotoxins that have been shown to cause neurological disorders in mammals, including muscle tremors and hyperexcitability in livestock, that have consumed feed contaminated with *A. flavus* (Valdes et al., 1985). β-aflatrem displayed significant activity against corn earworm in feeding studies (TePaske et al., 1992). Biosynthesis of aflatrems proceeds much like that of paxilline in *Penicillium paxilli* (Parker and Scott, 2004), in that aflatrem consists of a paxilline-like core, with an additional prenyl group on the indole moiety and an acetyl group on the diterpene skeleton (Nicholson et al., 2009). Utilizing sequence information for genes involved in paxilline biosynthesis in *P. paxilli* and the genome sequence of *A. flavus*, the genes required for aflatrem biosynthesis were found to be present on two separate loci in *A. flavus* (Zhang et al., 2004; Nicholson et al., 2009). Expression of aflatrem cluster genes and concomitant production of aflatrem has been shown to require the presence of *veA* and *laeA* (Duran et al., 2007; Georgianna et al., 2010).

Two gene clusters involved in aflatrem biosynthesis have been described. The ATM1 locus, present on chromosome 5 in *A. flavus*, harbors a gene cluster consisting of the *atmG, atmC,* and *atmM* genes. These encode the geranylgeranyl pyrophosphate (GGPP) synthase, prenyltransferases, and monoxygenase, respectively, that are involved in synthesis of paspaline, the first stable intermediate in paxilline and aflatrem biosynthesis. The ATM2 locus, located on chromosome 7, contains *atmD*, encoding an aromatic prenyltransferse; *atmQ* and *atmP,* both encoding P450 monooxygenases; and *atmA* and *atmB*, both encoding predicted membrane proteins believed (but not proven) to be transporters required for paxilline biosynthesis. The exact functions of *atmG*, *atmC*, *atmM*, and *atmB* and their pax orthologs in paspaline biosynthesis are not clear. It is believed that AtmG catalyzes the condensation of indole-3-glycerol phosphate and DMAPP to generate GGPP, which is then epoxidated by AtmM and cyclized by AtmC to form paspaline (Saikia et al., 2006). AtmP converts paspaline to 13-desoxypaxilline via removal of the C-30 methyl group and oxidation at C-10. AtmQ catalyzes the oxidation of 13-desoxypaxilline at C-7 then C-13 to form paspalinine. Finally, AtmD prenylates paspalinine on the indole moiety to form aflatrem. No pathway-specific transcription activator gene was identified in the clusters.

### CYCLOPIAZONIC ACID

It was noted that *A. flavus* strains unable to form AFs, due to deletions that extended from the adjacent subtelomeric region to within the AF gene cluster, were often unable to produce CPA (Chang et al., 2009). A region spanning about 30 kb from the subtelemeric end of the AF cluster was shown to harbor genes encoding a putative DMAT (*dmtA*), PKS-NRPS (*pks-nrps*), and FAD-dependent oxidoreductase (*moaA*) that were considered candidates for CPA production based on enzymes identified in biosynthesis of EAs. Inactivation of these three genes resulted in loss of CPA production. Orthologous genes (*cpaD = dmtA*; *cpaA* = *pks-nrps*; *cpaO* = *moaA*) have been identified in *A. oryzae* and also shown, by gene disruption, to be required for biosynthesis of CPA (Shinohara et al., 2011). Interestingly, the CPA cluster in both of these fungi also contained a putative transcription factor (*cpaR* = *ctfR1*), however, disruption of this gene in both *A. flavus* and *A. oryzae* did not affect CPA production. Production of CPA has been shown to require the presence of the *veA* gene (Duran et al., 2007).

In the initial step in CPA biosynthesis, the PKS-NRPS catalyzes the condensation of L-tryptophan and two molecules of acetyl-CoA to generate cycloacetoacetyl-L-tryptophan (cAATrp) which is then converted by the DMAT to β-CPA. The FAD-dependent oxidoreductase is then responsible for the cyclization of β-CPA to CPA (Shinohara et al., 2011). Interestingly, *A. oryzae* RIB40, which does not make CPA, was found to have a truncated version of the PKS-NRPS (*cpaA*) gene.

## CONCLUSION AND FUTURE PERSPECTIVES

It will be difficult to ascertain the exact role of individual sclerotial secondary metabolites in fungal biology. However, observations such as the induction of orsellinic acid production in *A. nidulans* upon co-culture with a soil microbe provide strong support for a role of these natural products in cross-species communication or defense against competing microbes. The potential role of sclerotial secondary metabolites as a chemical defense against insect predators is supported by the plethora of studies that have demonstrated their antiinsectan/antifeedant properties (Gloer, 1995, 2007). The finding of Cary et al. (2014) of preferential feeding by insects on sclerotia collected from a mutant *A. flavus* no longer producing asparasone A represents the first *in vivo* experimental evidence of the contribution of a secondary metabolite to sclerotial chemical defense. These types of gene knockout experiments should prove invaluable in identifying the contribution of individual secondary metabolites to fungal chemical defense. This will be important as many of the secondary metabolites identified in *A. flavus* have not been assigned to a predicted gene cluster, and it is highly probable that in the near future many of these orphan clusters will be found to produce compounds that are associated with the sclerotium. Advances in functional genomics and metabolomics will invariably accelerate the pace in the identification of secondary metabolic gene clusters associated with the synthesis of sclerotial compounds. Accordingly, these studies will also provide relevant information on the genetic regulatory networks governing activation and modulation of secondary metabolic gene clusters that play a role in sclerotial biology as well as other cellular processes. In this regard, continued structural and comparative analyses of sequenced fungal genomes, coupled with ever-increasing understanding of the molecular and functional biology of secondary metabolites in the Aspergilli, will undoubtedly accelerate the identification and functional characterization of secondary metabolite gene clusters in other filamentous fungi.

The majority of studies on the biological functions of sclerotial secondary metabolites have focused on their role in chemical defense against insect predators and other competing organisms. More research is needed to investigate other possible roles for these metabolites in sclerotial biology. For example, no information exists as to why *A. flavus* produces two morphotypes of sclerotia and if there is any difference in the secondary metabolic profiles of these morphotypes. If a metabolite(s) is consistently present in one sclerotial morphotype versus the other this may indicate a role for the metabolite(s) in sclerotial morphogenesis. Not only are S morphotype sclerotia smaller than L morphotype, but they are almost always produced in greater numbers. The ability to produce greater numbers of S morphotype sclerotia may represent an adaptive response by the fungus to survive insect predation compared to that of L strains. A correlation between selective pressure, due to predation, and sclerotial size has been suggested (Wicklow, 1988), in which long-term survival of a fungus is improved by the production of larger, better chemically defended sclerotia compared to those of fungi that produce numerous small sclerotia. However, it can also be argued that smaller size may increase the ease with which S morphotype sclerotia are damaged/consumed by predators and therefore the fungus has evolved to produce increased numbers as a means to ensure dissemination and survival of the species. During the course of evolution, selective pressure from increased predation on S morphotype sclerotia may have led to an increase in the levels/classes of antiinsectan secondary metabolites present in small sclerotia. The study of Chang et al. (2002) demonstrated that an increase in AF intermediates led to smaller sclerotial size in *A. flavus*. A similar correlation may be used to explain the existence of the S morphotype. If sclerotia of S morphotype *A. flavus* strains have increased levels of secondary metabolites compared to L morphotype, the increased demand for carbon building blocks (e.g., acetate) for biosynthesis of the additional secondary metabolites would result in less availability of carbon for sclerotial biogenesis, resulting in the observed small sclerotial morphotype. Chemical analysis of sclerotial extracts coupled with insect feeding studies should be able to shed some light on the relationship of sclerotial morphotype and fungivory.

Another unexplored area is the potential role of secondary metabolites in mating of normally asexual species of Aspergilli. It will be of interest to determine if secondary metabolite profiles differ in the stromata generated from the mating of two *A. flavus* strains compared to that present in the sclerotia produced during normal growth of each strain. Perhaps a chemical signal produced by hyphae of the interacting mating types can induce production of novel secondary metabolites in the sexual stromata that are not present in sclerotia of the individual mating partners. The chemical signal itself could be the product of a secondary metabolic gene cluster that is activated upon interaction of hyphae of opposite mating types. The presence of novel secondary metabolites in stromata would indicate that these compounds may play a role in the recognition and initiation of sexual reproduction by strains of opposite mating type, or they may be produced as a means of expanding the chemical arsenal of antiinsectan agents present in the fruiting structures.

Sclerotia are very important to the survival and dissemination of fungi in nature, and as such should be the target of strategies for control of fungal contamination of food and feed crops. As presented in this review, a number of global regulators that control production of secondary metabolites also control sclerotial formation. Novel technologies such as host-induced gene silencing can take advantage of host plant-derived siRNAs that target expression of these global regulators in the invading fungus. For example, maize can be transformed with RNAi-based constructs that generate siRNAs targeting *veA* or *nsdC* gene transcripts of *A. flavus*. This approach, in theory, would reduce both AF and sclerotial production in the invading fungus (Nunes and Dean, 2012). The soundness of this concept has already been demonstrated for control of several cereal pathogens, including barley powdery mildew (Nowara et al., 2010), wheat leaf rust (Panwar et al., 2013) and maize ear-rot caused by * F. verticillioides* (Tinoco et al., 2010). Continued study of the biogenesis and function of fungal secondary metabolites and their association with development, as well as elucidation of the regulatory mechanisms controlling production of these natural products, will facilitate the design of additional strategies to reduce the detrimental effects of pathogenic fungi.

## Conflict of Interest Statement

The Review Editor Geromy G. Moore declares that, despite being affiliated to the same institution as author Jeffrey W. Cary, the review process was handled objectively and no conflict of interest exists.
